# A Case Report of Longitudinal Extensive Transverse Myelitis: Ruling Out the Known Perpetrators

**DOI:** 10.7759/cureus.95095

**Published:** 2025-10-21

**Authors:** Majid Khan, Azeem Khalid, Sajid Ali, Aasima J Khan, Rezaur R Reza, Jose A Cardenas

**Affiliations:** 1 Internal Medicine, Aiken Regional Medical Centers, Aiken, USA; 2 Internal Medicine, Khyber Girls Medical College, Peshawar, PAK; 3 Neurology, Aiken Regional Medical Centers, Aiken, USA

**Keywords:** letm, longitudinally extensive transverse myelitis, neuromyelitis optica, systemic steroids, transverse myelitis

## Abstract

Transverse myelitis (TM) is a rare, heterogeneous syndrome described as acute or subacute inflammation of the spinal cord, and longitudinally extensive transverse myelitis (LETM) is the term used to describe TM that is severe enough to produce T2-weighted high signal intensity on sagittal spinal magnetic resonance imaging (MRI) spanning three or more vertebral segments. We present a case of a 40-year-old African American woman who presented with a 2-day history of progressive bilateral hand weakness. Neurological examination revealed bilateral hand and wrist weakness, more pronounced on the left, with paresthesia from the mid-forearms down, not following a dermatomal pattern. Basic lab work was within normal limits. Autoimmune and infectious workup, including antineutrophil cytoplasmic antibodies (ANCA), thyroid peroxidase (TPO) antibodies, West Nile virus IgG/IgM, and rapid plasma reagin (RPR), was unremarkable. Erythrocyte sedimentation rate (ESR) was elevated at 46. Antinuclear antibodies (ANA) comprehensive plus profile was negative except for positive ribonucleoprotein (RNP) antibodies. Cerebrospinal fluid (CSF) analysis showed no oligoclonal bands. Computed tomography (CT) scan of the head and MRI of the brain were normal. MRI of the cervical and thoracic spine revealed patchy, irregular increased signal from C4-C7. The patient was diagnosed with LETM. She was started on IV methylprednisolone for three days, followed by tapering doses of oral prednisolone, and reported 80% resolution of paresthesia and improved upper limb motor function. At the two-week neurology follow-up, the patient reported near-complete recovery. Repeat cervical spine MRI showed marked improvement with no new enhancement. Despite being uncommon, LETM has devastating clinical outcomes, and many patients suffer from severe disability if treatment is delayed. In order to achieve maximally beneficial outcomes and, in some situations, initiation of the right treatment to avoid further bouts of central nervous system (CNS) inflammation, early identification and the etiology of LETM are crucial.

## Introduction

Transverse myelitis is a rare, heterogeneous syndrome described as acute or subacute inflammation of the spinal cord, presenting as sensorimotor impairment below the level of injury and bowel/bladder dysfunction. Generally, it occurs independently and traverses the spinal cord, resulting in bilateral deficiencies. Longitudinally extensive transverse myelitis (LETM) is the term used to describe TM that is severe enough to produce T2-weighted high signal intensity on sagittal spinal magnetic resonance imaging (MRI) spanning three or more vertebral segments [1]. LETM encompasses a wide range of differential diagnoses, and its presence should initiate a thorough clinical evaluation and paraclinical testing that includes imaging results as well as the timing of symptom onset, the existence or lack of prior neurological symptoms, and laboratory studies for serum and cerebrospinal fluid (CSF) [2]. Although rare, LETM can lead to devastating outcomes, with many patients developing significant disability if treatment is delayed. Prompt recognition and determination of its underlying cause are essential to optimize outcomes and, in some cases, to initiate appropriate therapy that can prevent further episodes of central nervous system (CNS) inflammation [3].

## Case presentation

A 40-year-old African American woman presented with a 2-day history of progressive bilateral hand weakness, heaviness, and paresthesia, described as decreased grip strength and tingling from the mid-forearms down, more pronounced on the left. She reported difficulty holding objects. Her medical history included well-controlled hypertension managed with amlodipine, losartan, and metoprolol. She has no significant family, psychiatric, or surgical history.

On examination, she appeared healthy, alert, and oriented, with normal vital signs. Neurological examination showed intact cranial nerves. Upper limb exam revealed bilateral hand and wrist weakness, more pronounced on the left, with paresthesia from the mid-forearms down, not following a dermatomal pattern. The remainder of the upper and lower limb exams, cerebellar testing, gait, and systemic examination were unremarkable. Lab work showed normal complete blood count (CBC) and comprehensive metabolic panel (CMP), except for a mildly elevated aspartate aminotransferase (AST) of 60. Electrolytes, creatine kinase (CK), troponin, thyroid-stimulating hormone (TSH), folate, vitamin B12, and lipid profile were normal (Table 1). Urine beta-human chorionic gonadotropin (β-hCG) was negative. Autoimmune and infectious workup, including antineutrophil cytoplasmic antibodies (ANCA), thyroid peroxidase (TPO) antibodies, West Nile virus immunoglobulin G/M (IgG/IgM), and rapid plasma reagin (RPR), was unremarkable. Erythrocyte sedimentation rate (ESR) was elevated at 46. Antinuclear antibodies (ANA) comprehensive plus profile was negative except for positive ribonucleoprotein (RNP) antibodies that can be either a transient finding or possible mixed connective tissue disease.

**Table 1:** Laboratory findings

LAB	RESULT	Reference Value
Complete Blood Count		
White Blood Cells (10e3/mcL)	4.53	3.3 - 10.7
Red Blood Cells (gm/dL)	13.20	11.60 - 15.50
Platelets (10e3/mcL)	311	147 - 425
Complete Metabolic Panel		
Glucose (mg/dL)	107	74 -106
Sodium (mmol/L)	138	137 -145
Potassium (mmol/L)	4.1	3.5 - 5.1
Creatinine (mg/dL)	0.7	0.52 - 1.25
Bilirubin (mg/dL)	0.4	0.2 -1.3
Alkaline Phosphatase (units/L)	78	38 - 126
Aspartate Aminotransferase (units/L)	60	15 - 46
Alanine Aminotransferase (units/L)	15	0 - 35
Others		
Thyroid Stimulating Hormone (milli intl units/L)	0.937	0.465 - 4.680
Folate (ng/mL)	>20	2.76 - 20
Vitamin B12 (pg/mL)	751	239 - 931
Creatine Kinase (units/L)	69	30 - 170

**Figure 1:**
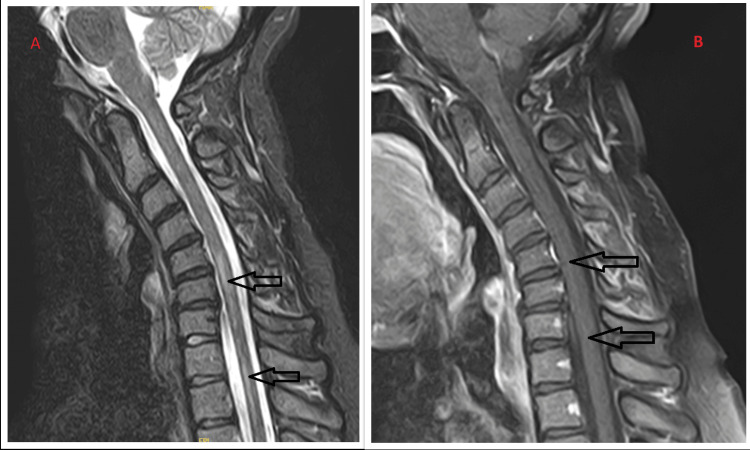
C-spine MRI (on presentation) 1A. Sagittal C-spine MRI with increased T2/STIR cord signal extending longitudinally from C5-C7 and C8-T1 (black arrows). Reversal of the anatomical cervical lordosis. Degenerative changes and disc bulges from C4-C7. 1B. Postcontrast T1 sagittal images revealed no abnormal enhancement within the cervical cord. C-spine: cervical-spine; STIR: short tau inversion recovery

The computed tomography (CT) scan of the head was unremarkable, with no evidence of hemorrhage, ischemia, or abnormal enhancement. The CT scan of the neck showed only reversal of cervical lordosis without fracture. Magnetic resonance imaging (MRI) of the brain with and without contrast was normal. MRI of the cervical spine revealed patchy, irregular, increased signal in the cervical and upper thoracic cord, suggestive of a demyelinating process. MRI of the thoracic spine confirmed abnormal signal from C5-T1; the rest of the thoracic spine was normal (Figure 1). EKG showed a normal sinus rhythm, and the chest X-ray was unremarkable. In view of cervical spine MRI, the patient was diagnosed with acute transverse myelitis, likely longitudinal extensive transverse myelitis (LETM). She was started on IV methylprednisolone 500 mg twice daily for three days and afterward put on tapering doses of oral prednisolone 60 mg, 40 mg, 20 mg, and 10 mg for 3 days each. She reported excellent improvement, with 80% resolution of paresthesia and improved upper limb motor function. Physical and occupational therapy evaluations found no ongoing needs, and she was discharged home.

**Figure 2:**
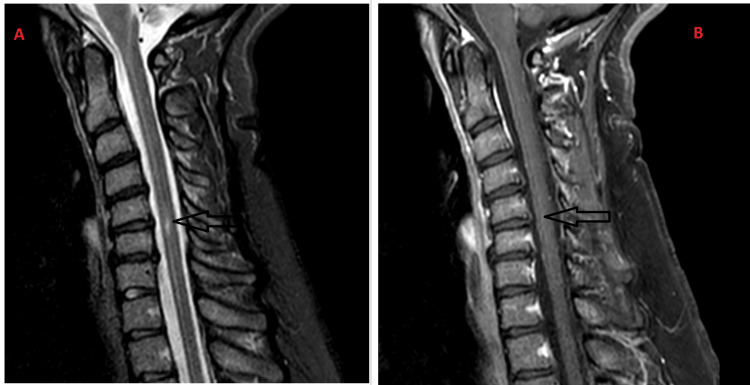
C-spine MRI (On Follow-up) 2A. Sagittal C-spine MRI with a focus of increased T2/STIR signal between C5-C6. Degenerative changes and disc bulges involving C4-C7, rectification of cervical cord, and improved reversal of cervical lordosis. 2B. Postcontrast T1 sagittal images with no abnormal enhancement within the cervical cord. C-spine: cervical-spine; STIR: short tau inversion recovery

At the two-week neurology follow-up, the patient reported near-complete recovery of her right hand, mild persistent weakness in the left hand, and new right leg numbness. There was no history of optic neuritis; therefore, neuromyelitis optica (NMO) antibodies were not tested. Due to ongoing paresthesia, a repeat cervical spine MRI and lumbar puncture were ordered. CSF analysis showed no oligoclonal bands, making multiple sclerosis unlikely. Repeat cervical spine MRI showed marked improvement with no new enhancement, consistent with a treated single episode of LETM (Figure 2). The patient was given a repeat prednisone tapering treatment for two weeks and followed up in one month. At one-month follow-up, the patient demonstrated excellent recovery. There is minimal numbness and occasional tingling in the right hand. At four-month follow-up, the patient had a near-complete recovery and no new or concerning neurological symptoms.

## Discussion

LETM is a condition caused by inflammation of the spinal cord, resulting in a mortality rate of 10% with 83% of survivors being ambulatory after a median of 12 years in the United States [4]. Based on clinical onset, LETM is categorized into 3 types: hyper-acute, evolving over minutes to four hours; acute/subacute, over 4 hours to 21 days; and chronic, over months [2]. It can affect both genders equally and can affect patients of all ages, but it has a bimodal peak between the ages of 10 and 19 and 30 and 39 [5]. Although the incidence of LETM is not well-studied, a retrospective review of 339 patients conducted in the USA has identified 2% cases of isolated idiopathic LETM. In a Brazilian study, 41 patients out of 70 were considered to have idiopathic acute TM, and 61% had radiological evidence of involvement of 3 or more vertebral segments. Sixty-five percent (65%) of the patients in a small Asian study involving 17 patients were reported to have longitudinally extensive T2 signals on MRI [6].

Etiologies of LETM can be broadly classified as idiopathic, postinfectious, systemic inflammation, or multifocal central nervous system disease, with the most common one being idiopathic [5]. LETM is a hallmark of neuromyelitis optica spectrum disorder (NMOSD) and is included in the revised diagnostic criteria of neuromyelitis optica (NMO) [7]. Multisystem autoimmune inflammatory disorders, particularly sarcoidosis, systemic lupus erythematosus (SLE), Sjögren syndrome, and Behcet disease, have an association with LETM [8]. Central nervous system (CNS) infections (e.g., syphilis, tuberculosis, Lyme disease, Varicella Zoster virus (VZV), HIV, Epstein-Barr virus (EBV), Cytomegalovirus (CMV), COVID-19, dengue fever, schistosomiasis, Toxocariasis) comprise additional causes of LETM. Paraneoplastic syndromes, primary spinal tumors, such as ependymomas and astrocytomas, and metabolic and vascular myelopathies have been reported to have an association with LETM [8].

MRI is the gold standard for diagnosing most spinal cord disorders, including LETM, as it confirms the diagnosis, excludes compressive myelopathy, and may suggest an underlying cause. Trebst et al. proposed a comprehensive initial workup for LETM, which includes detailed history-taking, clinical examination, whole-spinal MRI with contrast, brain MRI to rule out asymptomatic lesions, cerebrospinal fluid (CSF) analysis, and blood work for autoimmune markers such as anti-AQP4 IgG (for NMOSD), anti-nuclear (ANA), anti-double-stranded DNA (anti-dsDNA), antiphospholipid, anti-extractable nuclear antigen (anti-ENA), Rheumatoid Factor (RF), pANCA, cANCA, and onco-neuronal antibodies. In cases with elevated CSF cell counts (>50 cells/µL), infectious aetiologies must be ruled out using CSF PCR for viruses and Mycobacterium, and serological tests for HIV, HTLV-1, syphilis, Lyme disease, and tuberculosis. Further investigations may be performed in the appropriate clinical setting, including serum vitamin B12 and vitamin E levels, serum copper and ceruloplasmin, neuro-ophthalmologic evaluation, paraneoplastic evaluation, prothrombotic evaluation, spinal angiogram, and salivary gland biopsy [9,10].

General considerations for the management of LETM involve supportive care, such as deep venous thrombosis (DVT) prophylaxis, respiratory status monitoring, and pressure sore prevention, and addressing pain and sphincter disturbances. Distinguishing between inflammatory and non-inflammatory causes is crucial, as non-inflammatory etiologies require targeted treatments such as antimicrobials for infectious causes, supplementation of vitamins and minerals for deficiencies, or surgery for dural fistulae. Inflammatory causes are more common, and when infection is unlikely, high-dose IV corticosteroids like methylprednisolone 2.5-3g spread over three to five days are typically initiated, followed by oral corticosteroids tapered over one to three months to prevent rebound symptoms. In severe cases with significant disability or sphincter involvement, plasma exchange (PEX) is recommended if steroids fail. While intravenous immunoglobulin (IVIG) is effective in some immune disorders, its role in LETM remains unproven [1].

## Conclusions

Despite being uncommon, LETM has devastating clinical outcomes, and many patients suffer from severe disability if treatment is delayed. In order to achieve maximally beneficial outcomes and, in some situations, initiation of the right treatment to avoid further bouts of central nervous system (CNS) inflammation, early identification and the etiology of LETM are crucial. Although a definitive underlying cause could not be identified, this case underscores the importance of considering rare idiopathic presentations and highlights the need for long-term follow-up to better understand their clinical course and outcomes.
